# Actual Persistence of Abatacept in Rheumatoid Arthritis: Results of the French-Ric Network

**DOI:** 10.3390/jcm9051528

**Published:** 2020-05-19

**Authors:** Jean-Hugues Salmon, Jean-Guillaume Letarouilly, Vincent Goëb, Lukshe Kanagaratnam, Pascal Coquerelle, Marie-Hélène Guyot, Eric Houvenagel, Nicolas Lecuyer, Laurent Marguerie, Gauthier Morel, Guy Baudens, Elisabeth Gervais, René-Marc Flipo

**Affiliations:** 1 Faculty of Medicine, University of Reims Champagne-Ardenne, EA 3797, 51092 Reims, France; lkanagaratnam@chu-reims.fr; 2 Rheumatology Department, Hôpital Maison Blanche, Reims University Hospital, CEDEX, 51092 Reims, France; 3 CHU Lille, Rheumatology Department, F-59000 Lille, France; jeanguillaume.letarouilly@gmail.com (J.-G.L.); rene-marc.flipo@chru-lille.fr (R.-M.F.); 4 Rheumatology Department, Hôpital Nord, University Hospital of Amiens-Picardie, 80000 Amiens, France; goeb.vincent@chu-amiens.fr; 5 Department of Research and Innovation, Robert Debre’ Hospital, Reims University Hospitals, 51092 Reims, France; 6 Rheumatology Department, Bethune Hospital Center, 62408 Bethune, France; pcoquerelle@ch-bethune.fr; 7 Rheumatology Department, Hôpital Victor Provo, Hospital of Roubaix, 59100 Roubaix, France; marie-helene.guyot@ch-roubaix.fr; 8 Rheumatology Department, Hôpital Saint Philibert, Hospital of Lomme, 59160 Lomme, France; houvenagel.eric@ghicl.net; 9 Rheumatology, Medical Office, Boulevard du Dr Schweitzer, 02100 Saint-Quentin, France; nicolaslecuyer02@gmail.com; 10 Rheumatology Department, Institut François Calot, 62600 Berck-Sur-Mer, France; lmarguerie@hopale.com; 11 Department of Rheumatology, Valenciennes Hospital Center, 59300 Valenciennes, France; morel-g@ch-valenciennes.fr; 12 Rheumatology, Medical Office, 39 Quai des Mines, 59300 Valenciennes, France; gbaudens@wanadoo.fr; 13 Department of Rheumatology, Poitiers University Hospital, 86021 Poitiers, France; e.gervais@chu-poitiers.fr

**Keywords:** rheumatoid arthritis, abatacept, persistence

## Abstract

Objectives: Data on abatacept (ABA) persistence in routine practice are limited. We aimed to study ABA persistence rates at 12 months, according to the date of initiation, and to analyze the factors associated with persistence at 12 months. Methods: We performed an observational, ambispective, multi-center study from January 2008 to July 2016, based on the French-RIC Network. We defined three groups of patients followed up for rheumatoid arthritis (RA), according to the date of initiation of ABA therapy: Group 1 (from 2007 to 31 July 2010: ABA indicated after anti-TNF failure); Group 2 (from 1 August 2010 to 31 March 2014: ABA indicated after conventional antirheumatic drugs failure); Group 3 (from 1 April 2014 to 1 July 2016: ABA available by the subcutaneous injection). Results: Among 517 patients who initiated ABA, drug persistence at 12 months was 68%. The only factor significantly associated with persistence rate at 12 months was C-reactive protein (CRP) < 10 mg/L at ABA initiation (odds ratio (OR) 0.6, 95% confidence interval 0.3–0.9; *p* = 0.0016). There was no significant difference in drug persistence according to date of initiation, the line of biological disease-modifying antirheumatic drugs (bDMARD) therapy or the route of administration. Conclusions: In routine practice, over time, ABA has come to be initiated earlier in the course of therapy for RA in France. Abatacept persistence is similar to that reported in the Orencia Rheumatoid Arthritis (ORA) registry, and does not differ according to the date of initiation. The only factor found to be associated with the persistence rate at 12 months was CRP < 10 mg/L at ABA initiation.

## 1. Introduction

Rheumatoid arthritis (RA) is the most common chronic inflammatory rheumatic disease [1,2]. It can cause substantial handicap, and can be life-threatening [1]. The therapeutic management of RA has been profoundly changed by the advent of biological agents (biologics). Initially, only TNF alpha inhibitors were available [3,4], but other drugs successively emerged, with different mechanisms of action and different therapeutic targets, such as anti-CD20 (rituximab) and a humanized (Tocilizumab) and the human (sarilumab) monoclonal antibody, recognizing the soluble and membrane-bound forms of IL-6 receptor [3,4]. Among available biologics, abatacept (ABA) is a recombinant protein that comprises the extracellular domain of human CTLA-4 fused to the Fc portion of human IgG1. Activated T cells are implicated in the pathogenesis of RA via amplification of the inflammatory cascade that leads to joint inflammation and destruction in RA. ABA acts by competing with CD28 for binding to CD80/CD86, modulating the second costimulatory signal required for full T-cell activation. In France, ABA is indicated for the treatment of RA since 2007 in association with methotrexate (MTX). However, its role in the therapeutic management of adult RA has changed over time. Initially, ABA was recommended as third-line biologic therapy (after anti-TNF therapy failure). Since 1 August 2010, it can be prescribed as second-line biologic therapy (conventional antirheumatic drugs failure including MTX), and since 1 April 2014, has been available for subcutaneous administration. 

Currently available data regarding the safety and efficacy of ABA stem mainly from clinical trials or industry-sponsored studies. While data regarding persistence on drugs exist for the TNF inhibitor, data on ABA use in routine practice are limited [5,6,7,8,9]. The lack of studies on abatacept has been recognized [10]. In France, only the Orencia Rheumatoid Arthritis (ORA) registry had sufficient data to study long-term safety and efficacy of ABA, outside the context of clinical trials, in patients treated for RA and followed up prospectively for seven years [11,12,13]. 

However, the patients in the ORA registry were included from 2008 to 2009. Therefore, the ORA data do not enable any analysis of the impact that the changing place of ABA in the therapeutic armamentarium may have had on drug persistence and safety. 

The main objective of this study was therefore to study ABA persistence. To this end, we used data from the RIC-France Network, a database of patients with chronic inflammatory RA. The secondary objectives were to identify the factors associated with drug persistence at 12 months, persistence rate according to the year of treatment initiation, the reasons for discontinuation of ABA therapy, and efficacy criteria. 

## 2. Methods 

### 2.1. Patient Selection

The RIC-France Network is a database of patients with chronic inflammatory RA. Inclusion has been ongoing since 2004, and patients are entered into the registry by rheumatologists participating in the registry, in the hospital setting or private practice. The Network was approved by the national commission for the protection of personal data (Commission nationale de l’informatique et des libertés, CNIL, No. 1653314 v. 1). All participants provided written informed consent.

We performed an ambispective, multicentric observational study based on the data from the RIC-France Network of patients with chronic inflammatory RA from January 2008 to July 2016.

Inclusion criteria for the patients were:

- RA according to the 2010 ACR/EULAR criteria [14];
- Age 18 years or older;
- Initiation of ABA therapy by the intravenous or subcutaneous route;
- Attendance at a minimum of 12-months follow-up;
- Written informed consent for participation in the RIC-France Network.

Non-inclusion criteria were refusal to participate and patients under legal protection measures.

### 2.2. Data Recorded

Data were collected as part of routine clinical practice and electronic medical records were obtained (e-CRF). For each patient, the following variables were recorded from the patient’s medical file: baseline characteristics of the patient (age, sex, weight, height, body mass index, comorbidities); disease characteristics (duration of RA, positivity of rheumatoid factor (RF) and anticitrullinated protein antibodies (ACPAs), presence of erosion, prior treatment, disease activity score in 28 joints using erythrocyte sedimentation rate (DAS-28 ESR) at treatment initiation, C-reactive protein (CRP) level at initiation, concomitant treatments). Data related to treatments taken in addition to the biologic, and relating to disease activity, were measured at 6 and 12 months, and when possible, at 24 months. In patients who discontinued ABA, the reasons for discontinuation were recorded. 

Drug persistence was defined as the continuation of abatacept treatment over time. The primary endpoint was the rate of ABA persistence at 12 months. 

We also sought to investigate the change in ABA’s line treatment in the therapeutic management of RA over time. To this end, we defined three patient populations:

- Group 1: patients who initiated ABA therapy from 2007 to 31 July 2010 (ABA indicated after anti-TNF alpha failure);
- Group 2: patients who initiated ABA therapy from 1 August 2010 to 31 March 2014 (ABA indicated after conventional antirheumatic drugs failure including MTX);
- Group 3: patients who initiated ABA therapy from 1 April 2014 to 1 July 2016 (ABA available by the subcutaneous injection) up to 1 July 2016.

Regarding the efficacy criteria for ABA, we used validated composite criteria, including joint indices [4]. Clinical remission was defined as a DAS28-ESR ≤ 2.6, and low disease activity as a DAS28-ESR ≤ 3.2 [15]. These criteria can overestimate clinical remission rates, as they may indicate a remission despite the persistence of clinical synovitis in one or more joints [15,16]. Consequently, the DAS28-ESR, SDAI, and CDAI seem to classify patients similarly for minimal disease activity but not for remission [17].

### 2.3. Statistical Analysis

Quantitative variables are expressed as mean ± standard deviation, and categorical variables as number (percentage). Drug persistence (“survival”) curves were plotted using the Kaplan–Meier method, and curves were compared using the log-rank test. 

Bivariable analysis was performed using logistic regression to identify the factors associated with drug persistence at 12 months. Multivariable analysis by logistic regression was performed including all variables with *p* < 0.20 by bivariable analysis. Results are expressed as odds ratios (OR) with 95% confidence intervals (CI). *p* < 0.05 was considered statistically significant. All analyses were performed using SAS version 9.4 (SAS Institute Inc., Cary, NC, USA). 

## 3. Results

### 3.1. Patient Selection and Characteristics

**Figure 1:**
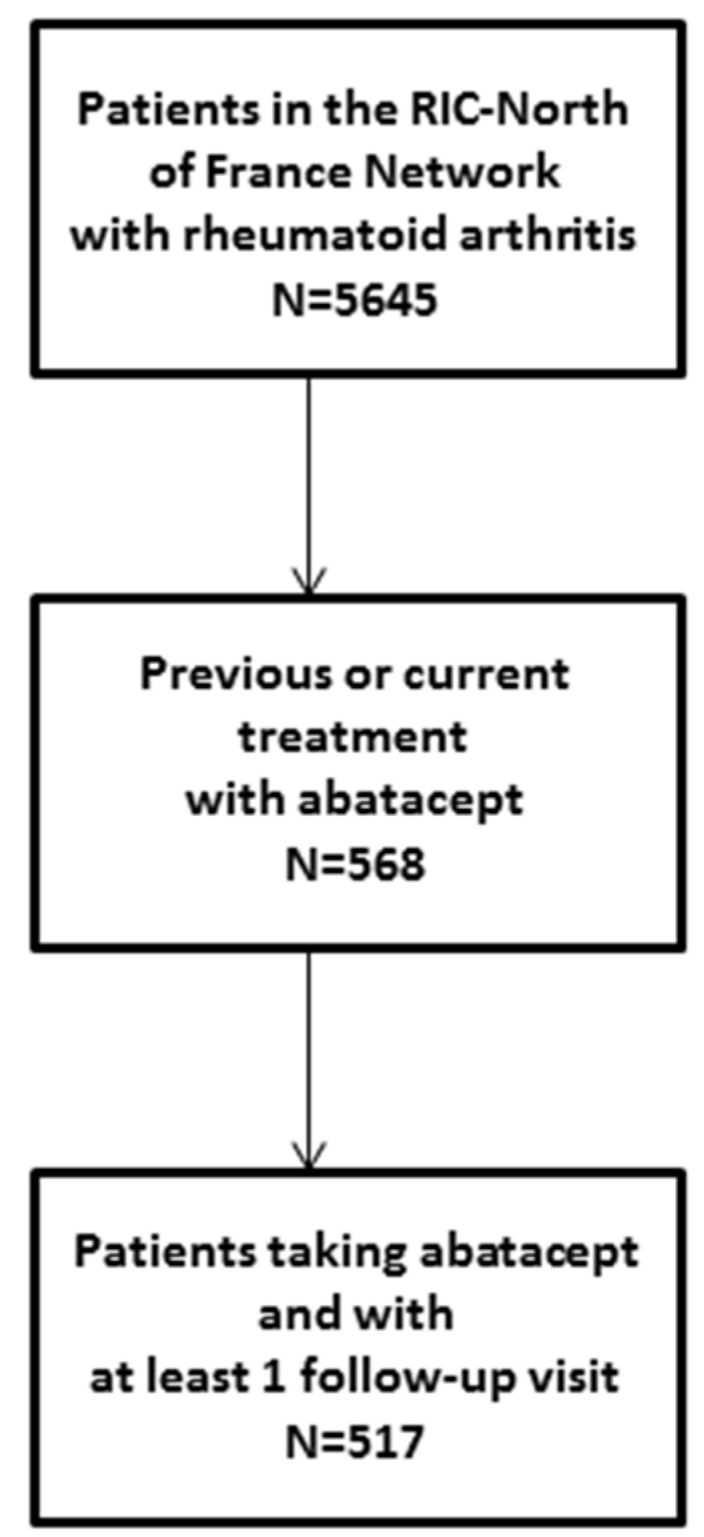
Flow chart of the study population.

Among 5464 patients followed for RA in the RIC-France Network, 517 met the inclusion criteria (Figure 1). The characteristics of the 517 patients are shown in Table 1. 

### 3.2. ABA Persistence in the Overall Population

**Figure 2:**
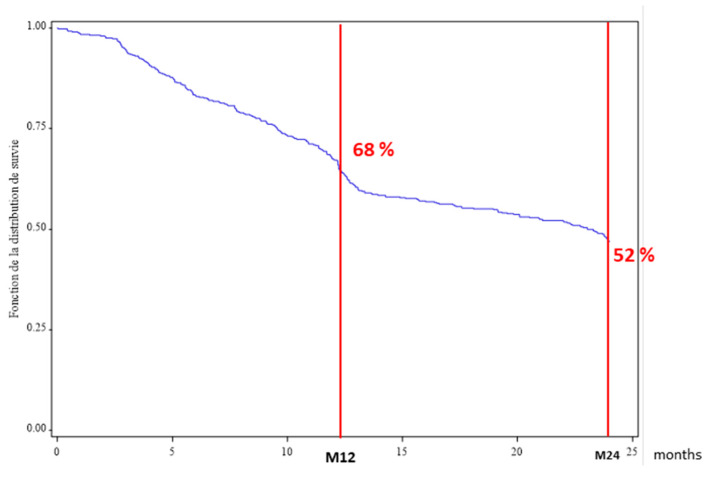
Abatacept persistence at 12 and 24 months.

According to the Kaplan–Meier curve (Figure 2), the rate of drug persistence at 12 months was 68%. Among 445 patients with a two-year follow-up, the rate was 52%. 

### 3.3. Reasons for ABA Discontinuation

At 12 months, 166 patients (32%) had discontinued ABA therapy. Treatment was discontinued on average after 23 ± 20.5 months of therapy, or a median of 21 (9.1–29.3) months. The reasons for ABA discontinuation were: primary inefficacy in 54 patients (32.5%), adverse effects in 44 (26%), secondary inefficacy in 40 (24%) and other reasons (pregnancy, surgery, loss to follow-up) in 28 (17.5%). 

### 3.4. Factors Associated with Drug Persistence at 12 Months 

By bivariable analysis, factors associated with drug persistence at 12 months were female sex, presence of RF, presence of erosion, lower DAS28 at initiation, and CRP < 10mg/L at initiation of ABA (Table 2). 

By multivariable analysis, there was a significant association between drug persistence at 12 months and CRP < 10 mg/L at ABA initiation (OR 0.6, 95%CI 0.3–0.9; *p* = 0.0016). 

No other demographic, clinical or paraclinical characteristics were found to be associated with drug persistence at 12 months. 

### 3.5. Efficacy Criteria

Average DAS28 ESR at ABA initiation was 4.7 ± 1.3 (*n* = 512). Among these patients, at initiation, 38 (7.5%) were in remission, 30 (6%) had low disease activity and 444 (86.5%) had active RA. At 12 months (*n* = 351), 106 patients (30%) were in remission, 64 (18.5%) had low disease activity, and 181 (51.5%) had active RA. At 24 months (*n* = 217), the rates were, respectively, 70 (32.5%), 45 (20.5%) and 102 (47%). 

### 3.6. Patient Characteristics According to Date of ABA Initiation 

The characteristics of the three groups of patients differed according to the date of ABA initiation (Table 3). Comparing the different periods of ABA initiation, in Group 3, ABA is initiated in younger patients with more recent RA (23 vs. 14 years), with less erosion (83% vs. 37%), and less active disease. The association with MTX is more frequent (27% vs. 58%). Furthermore, ABA is prescribed earlier in the management strategy (9% vs. 31%). The period of initiation was not associated with a significant difference in the rate of drug persistence. 

### 3.7. Relation between Drug Persistence and Other Factors 

There was no significant difference in drug persistence according to the line of biological disease-modifying antirheumatic drugs (bDMARD) therapy, the route of administration or the date of initiation of ABA.

## 4. Discussion

Our study evaluated ABA persistence in patients with RA, in routine practice over a long period (2007–2016), with, respectively, 83%, 68% and 52% continuing treatment at 6, 12 and 24 months. The only factor found to be significantly associated with drug persistence at 12 months was CRP < 10 mg/L at ABA initiation (OR 0.6, 95%CI 0.3–0.9; *p* = 0.0016). Finally, ABA seems to be introduced increasingly early in the management of RA in France.

Regarding drug persistence, our results at 12 months are comparable to those of the observational ACTION study (*n* = 2350), which reported pursuance of intravenous ABA in 78% in bDMARDS naïve patients, and in 70% of patients with failed bDMARD therapy [18]. Conversely, drug persistence at 24 months was similar, at 48% [19,20]. 

In a retrospective, multicenter study from Japan (ANSWER), 681 patients received ABA for RA, of whom 60% were biologic-naïve. The rate of drug persistence at 36 months was 75.5% [21]. This trend was confirmed in the ASCORE study of subcutaneous ABA, with a persistence rate of 65% at one year. The rate was higher in bDMARDS naïve patients (71.1%) as compared to patients who failed bDMARDS (one bDMARDs 61.9%; ≥ 2 bDMARDs: 60.7%) [22]. One possible explanation for these differences could be the low number of bDMARD-naïve patients in our cohort (22%). 

The PAN-ABA study pooled data from nine European observational cohorts of patients treated with ABA, with the median year of ABA initiation in 2009. The median number of bDMARDS prior to ABA initiation varied from one to two. In this study, wide variations in ABA persistence rates were shown across European countries [9]. Indeed, at 12 months, ABA persistence varied from 50% to 85% across countries. The reasons proposed to explain this heterogeneity included differences in patient and disease characteristics, but also differences in national economic characteristics [9]. In many countries, high drug-costs still limit widespread use and thus contribute to inequity of access to the best care. This inequity access to bDMARDs across Europe can have an impact on drug retention [9]. It should be noted that our results are similar to those reported by the French ORA registry (Orencia and Rheumatoid Arthritis) [9,13]. 

Studies comparing persistence rates and safety of biologics in daily routine practice are sparse, and mainly focused on anti-TNF alpha agents [23]. Based on data from the French national health insurance database, it was shown that almost half of patients with inflammatory rheumatic disease discontinued subcutaneous anti-TNF alpha treatment after one year [24].

Recently, a French study compared the efficacy and safety of rituximab, ABA and tocilizumab in adults with RA and inadequate response to TNF inhibitors in three French Society of Rheumatology registries. Treatment with rituximab or tocilizumab was associated with a greater improvement in outcomes at two years compared to ABA [25]. ABA persistence at 24 months was lower than that observed in our study (39.3% vs. 52% in our cohort) [25]. Conversely, in the ANSWER study, the persistence rate with ABA was higher than for other bDMARDS [21].

In our study, the only factor found to be associated with higher drug persistence was CRP < 10 mg/L at ABA initiation. Contrary to the data from the PAN-ABA and ACTION studies, the number of prior bDMARDS and the serological status were not found to be associated with ABA persistence [5,18,20]. There are differences to be taken into account in interpreting and comparing our results with previous studies. For example, patients that could receive abatacept as first-line biologic, have 18 and 14 years of mean disease duration, respectively. Most patients were not onset recent RA or bDMARD-naïve.

The reason for ABA discontinuation in our study was the loss of efficacy or primary failure. As previously reported in the literature, discontinuation due to adverse effects, notably infectious events, was less frequent [21,22,25].

The main originality of this study is to have data on three different prescription periods. Since 2014, ABA has been initiated earlier in the therapeutic strategy with more patients on first-line biologic treatment and in more recent RA. The mean DAS 28 at initiation is lower, reflecting the current trend to initiate bDMARDs earlier in the treatment strategy and in less active RA. Abatacept in combination with MTX was more common: Whereas it is recommended to use bDMARDs in combination with csDMARDs. In current practice, 40% of patients are on monotherapy [26]. The hypotheses to explain prescriptions of biologic agents as monotherapy include previous adverse reactions to csDMARDs resulting in gastrointestinal side effects, rashes, blood dyscrasias or hepatotoxicity, while for other patients had more comorbidities (a typically more challenging group to treat) or csDMARDs were contraindicated.

Our study has several strengths, as well as some limitations. The main limitation is the retrospective nature of the study, but this approach enables us to study real-life data and avoid potential overestimations. This was an observational study, with the inherent risk of missing data, loss to follow-up, and a lack of a control group. The relatively small sample size may mean that our study is underpowered, although, with a total of 517 patients, our study population is larger than the average in the PAN-ABA countries. Finally, there is likely some potential for indication bias, since ABA may have been prescribed preferentially in certain patients. However, data on comorbidities and changes in ACPA levels [27], which might influence drug survival and treatment response, were not available.

The main strength of this study lies in the selection and representativeness of the patient population. Indeed, this was a large, multicenter cohort, bringing together patients seen in both the hospital and private practice settings. This enables our study to provide a true picture of daily routine practice. Finally, the long study period (2007 to July 2016) made it possible to assess the impact of changes in ABA’s line treatment among the therapeutic armamentarium for RA. 

## 5. Conclusions

In conclusion, in patients treated for RA with ABA in routine practice, ABA appears to be introduced increasingly early in the therapeutic management of RA over the course of time in France. Drug persistence is similar to that reported in the ORA registry, and does not differ significantly according to the year of ABA initiation. The only factor found to be associated with greater drug persistence at 12 months was CRP < 10 mg/L at treatment initiation. 

## Figures and Tables

**Table 1:** Baseline characteristics of the study population (*n* = 517).

	Whole Population	Available Data
Age	61.4 ± 13.3	517 (100%)
Female sex	381 (73.7%)	517 (100%)
Disease duration (years)	18.1 ± 10.6	503 (97%)
Body mass index	26.9 ± 6	467 (90%)
RF positive	365 (77.9%)	468 (90.5%)
ACPA positive	347 (76.4%)	454 (88%)
Erosion	327 (70.9%)	461 (89%)
DAS 28 at initiation	4.7 ± 1.3	512 (99%)
C-reactive protein	17.6 ± 25	406 (78.5%)
Erythrocyte sedimentation rate	27.7 ± 22.3	440 (85%)
Methotrexate at initiation	228 (44.1%)	517 (100%)
-dose in mg/w	14.2 ± 4.7	
Corticosteroids at initiation	212 (41%)	517 (100%)
-dose in mg/d	9.1 ± 5.6	
Mean number of prior conventional DMARDs	1.5 ± 1.3	517 (100%)
Mean number of prior bDMARDs	1.6 ± 1.2	517 (100%)
0	114 (22%)	
1	145 (28%)	
2	149 (29%)	
≥3	109 (21%)	

RF, rheumatoid factor; ACPA, anticitrullinated protein antibodies; DAS28: disease activity score in 28 joints; DMARDs: disease-modifying antirheumatic drugs; b, biologic.

**Table 2:** Factors associated with drug persistence at 12 months.

	Persistence < 12 Months *n* = 166	Persistence ≥ 12 Months *n* = 351	Bivariable Analysis OR (95% CI)	Multivariable Analysis OR (95% CI)
Age	60.8 ± 13.5	61.6 ± 13.2	1.1 (0.70–1.60)	
Female sex	113 (68%)	268 (76%)	1.5 (1–2.30)	1.6 (0.90–2.70)
Disease duration (years)	18.4 ± 11	17.9 ± 10.5	1 (0.90–1.10)	
Body mass index	26.8 ± 5.9	27 ± 6	0.9 (0.60–1.60)	
RF positive	111 (74%)	254 (80%)	1.4 (0.90–2.20)	1.1 (0.60–1.90)
ACPA positive +	105 (73%)	242 (78%)	1.3 (0.80–2.10)	
Erosion	98 (67%)	229 (73%)	1.3 (0.90–2.10)	1.5 (0.90–2.40)
DAS 28 at initiation	4.9 ± 1.3	4.5 ± 1.4	0.3 (0.10–0.80)	0.3 (0.10–1.10)
CRP < 10 mg/L at initiation	109 (68%)	197 (43%)	0.5 (0.40–0.80)	0.6 (0.30–0.90)
ESR	32.1 ± 25.3	25.5 ± 20.3	0.98 (0.97–0.99)	0.99 (0.98–1.10)
MTX at initiation	72 (43%)	156 (44%)	1 (0.70–1.50)	
Corticosteroids at initiation	93 (56%)	212 (60%)	0.8 (0.50–1.20)	
Mean number of prior conventional DMARDs	1.6 ± 1.4	1.4 ± 1.3	0.9 (0.70–0.90)	0.9 (0.70–1.10)
Mean number of prior bDMARDs	1.6 ± 1.2	1.6 ± 1.3	0.8 (0.50–1.40)	

RF, rheumatoid factor; ACPA, anticitrullinated protein antibodies; DAS28: disease activity score in 28 joints; CRP, C-reactive protein; ESR, erythrocyte sedimentation rate; MTX, methotrexate; DMARDs: disease-modifying antirheumatic drugs; b, biologic.

**Table 3:** Patient characteristics according to the date of abatacept initiation.

	Before 1 August 2010 (Anti-TNF Failure) *n* = 137	1 August 2010–31 March 2014 (First-Line Possible) *n* = 175	After 1 April 2014 SC Route Possible *n* = 205
Age	65.4 ± 11.7	61.5 ± 12.3	58.5 ± 14.4
Disease duration (years)	23.3 ± 10.5	18.9 ± 9.6	14 ± 10
Erosion at initiation	101 (83%)	101 (57.7%)	76 (37%)
DAS 28 at initiation	5.3 ± 1.2	4.7 ± 1.2	4.2 ± 1.4
CRP < 10 mg/L at initiation	29 (27.3%)	74 (42.3%)	72 (35.1%)
MTX at initiation	32 (27.5%)	76 (43.4%)	120 (58%)
Corticosteroids at initiation	44 (32%)	75 (43%)	93 (45%)
Prior conventional DMARDs			
-mean ± SD	1.5 ± 1.3	1.4 ± 1.2	1.4 ± 1.3
-median (IQR)	1 (0–3)	1 (0–2)	1 (0–2)
Prior bDMARDs			
0	12 (9%)	39 (22%)	63 (31%)
1	30 (22%)	58 (33%)	57 (28%)
2	51 (37%)	43 (25%)	55 (27%)
≥3	44 (32%)	35 (20%)	30 (14%)
-mean ± SD	2.1 ± 1.2	1.5 ± 1.3	1.3 ± 1.2
-median (IQR)	2 (1–3)	1 (1–2)	1 (0–2)

SC, subcutaneous; DAS28: disease activity score in 28 joints; CRP, C-reactive protein; MTX, methotrexate; DMARDs: disease-modifying antirheumatic drugs; b, biologic; SD, standard deviation; IQR, interquartile range.
